# Longitudinal changes in the retail food environment in Mexico and their association with diabetes

**DOI:** 10.1016/j.healthplace.2020.102461

**Published:** 2020-11

**Authors:** Carolina Pérez-Ferrer, Amy H. Auchincloss, Tonatiuh Barrientos-Gutierrez, M. Arantxa Colchero, Leticia de Oliveira Cardoso, Mariana Carvalho de Menezes, Usama Bilal

**Affiliations:** a National Institute of Public Health, Avenida Universidad 655, Santa María Ahuacatitlán, 62100, Cuernavaca, Mexico; b National Council for Science and Technology (CONACYT), Mexico; c Dornsife School of Public Health, Drexel University, Philadelphia, PA, USA; d Fiocruz-RJ, Brazil, Av. Brasil, 4365, Manguinhos, Rio de Janeiro, Brazil; e Federal University of Ouro Preto, R. Dois, 607, 35400-000, Ouro Preto, Brazil

**Keywords:** Built environment, Food supply, Type 2 diabetes mellitus, Mexico, Urban health, Convenience foods, Supermarkets

## Abstract

The retail food environment is a potential population-level determinant of diet and nutrition-related chronic diseases, yet little is known about its composition and association with diabetes in low- and middle-income countries. Our objectives were: (1) to describe changes in the composition of the retail food environment in Mexican neighborhoods from 2010 to 2016 and (2) to examine the association between these changes and diabetes cases diagnosed over the same period. Individual level data came from the 2016 Mexican Health and Nutrition Survey (N = 2808 adults). Neighborhood level retail food environment data for 2010 and 2016 came from the National Directory of Economic Units of Mexico. Multilevel logistic regression was used to examine the adjusted association between changes in the neighborhood density per km^2^ of fruit and vegetable stores, chain convenience stores and supermarkets with diabetes. Small store formats still predominate in Mexico's food environment, however there is evidence of fast increase in chain convenience stores and supermarkets. Adults living in neighborhoods that saw a decline in fruit and vegetable store density and a simultaneous increase in chain convenience store density experienced higher odds of diabetes, compared to adults who lived in neighborhoods where fruit and vegetable and convenience stores did not change (OR 3.90, 95% CI 1.61, 9.48). Considering the complex interplay between store types, understanding the mechanisms and confirming the causal implications of these findings could inform policies that improve the quality of food environments in cities.

## Highlights

• We examined changes in the retail food environment in Mexican cities.
• We also examined whether those changes were associated with new diabetes cases.
• Small food stores still predominate but evidence of fast increase in chain convenience stores and supermarkets.
• Odds of diabetes were highest when fruit and vegetable stores declined and chain convenience stores increased.
• Changes in the density of supermarkets were not associated with diabetes.

## 1 Introduction

The burden of diabetes type 2 in the Latin American region is high and increasing (Rojas-Martinez et al., 2018; World Health Organization, 2011). Specifically, the prevalence of diabetes in Mexico in 2016 was 13.7% (Basto-Abreu et al., 2020). This burden is linked to rising obesity prevalence and changes in diet such as higher consumption of refined carbohydrates, added sweeteners and edible oils (Imamura et al., 2015; Popkin, 2015). The retail food environment, i.e. where people shop for their food, is a potential population-level determinant of an individual's diet and diet-related chronic diseases. The mix of food outlets in a local area determines the type of foods available to be purchased and consumed. For example, a higher availability of fruit and vegetable stores has been associated with higher consumption of fruits and vegetables which in turn could be preventive of diabetes (Duran et al., 2016; Qian et al., 2019). On the other hand, a higher availability of stores which sell mainly ultra-processed foods and beverages may increase the risk of diabetes through higher consumption of ultra-processed foods (Imamura et al., 2015). The food environment may also be linked to diabetes indirectly through the effect of these dietary behaviors on body mass index (Imamura et al., 2015; Schwingshackl et al., 2017) (Supplementary Fig. 1). Therefore, the retail food environment is a potential modifiable factor that may help in reducing diabetes burden (Glanz et al., 2005).

The food environment in Latin American cities is characterized by the predominance of small retailers over large chains. Small retailers include fruit and vegetable stores and other specialized stores which sell almost exclusively fresh produce and do not sell ultra-processed foods. However, the food environment in this region is experiencing significant changes concurrent with the increase in obesity and diabetes prevalence. In Mexico, for example, the grocery and convenience store markets are growing approximately 10 percent per year, with smaller supermarkets and chain convenience stores being the fastest growing sectors in retail (Deloitte Planet Retail, 2017; USDA Foreign Agricultural Service, 2016). In the convenience store sector, this growth has been fueled mostly by outlets of the chain OXXO (over 11,000 outlets or nearly 80% of the market (Mexico News Daily, 2014),) and 7-eleven (over 1800 outlets) (7-Eleven Corporate, 2016). Chain convenience stores sell mostly ultra-processed foods and beverages. It is not clear to what extent supermarkets and chain convenience stores are displacing smaller stores selling healthy food. In Spain, areas with increasing growth of supermarkets have seen a decrease in food and vegetable stores and other food stores that do not sell ultra-processed foods (Bilal et al., 2018b).

The association between the food environment and obesity and diabetes has been studied repeatedly in high-income countries (Bilal et al., 2018a; Cobb et al., 2015). In a systematic review of the literature, there was weak to null evidence on the association between grocery stores or supermarkets and lower diabetes risk (Bilal et al., 2018a). However, diabetes incidence and prevalence was found to be higher in people who live in, or move to areas with a higher density of convenience stores and fast-food outlets (Christine et al., 2015; Gebreab et al., 2017; Mezuk et al., 2016). Little work has been done to assess the contribution of the retail food environment to obesity and diabetes in Latin America (Perez-Ferrer et al., 2019). Research in this context is necessary because of the unique characteristics of the food environment (i.e. high density of fresh produce stores) which may be protective against nutrition related chronic diseases, but also because of the fast changes in the environment which may be having a negative effect on health. Understanding the health effect of growth in convenience stores and supermarkets against potentially decreasing numbers of fresh produce stores is crucial for planning place based interventions.

Another aspect to consider when studying the food environment and its effect on health is that it may not affect all individuals equally. More socially advantaged groups may be in a position to travel further distances to buy their choice of food. Further, education is associated with dietary choices (Hinnig et al., 2018) so individuals exposed to the same retail food environment could chose different types of food depending on their education level. Education level may therefore act as a buffer to unhealthy food environments. There is evidence that education is inversely associated with obesity and diabetes especially among women in low and middle income countries (Hinnig et al., 2018; Pérez-Ferrer et al., 2018; Rojas-Martinez et al., 2018). To our knowledge, no study in Latin America has assessed the association of the retail food environment with diabetes, the role of changes in the food environment in nutrition-related diseases, the role of education as a potential effect modifier of this association, or has looked at more than one city or a few neighborhoods (Duran et al., 2016; Jaime et al., 2011; Mendes et al., 2013; Molina et al., 2017; Pessoa et al., 2015).

Therefore, in this study our objectives were: (1) to describe the changes in the composition of the retail food environment in Mexican cities from 2010 to 2016, using an economic census of Mexico (Instituto Nacional de Geografía y Estadística, 2018); and (2) to examine the association between changes in the composition of the retail food environment, specifically fruit and vegetable stores, chain convenience stores and supermarkets, from 2010 to 2016 and diabetes cases diagnosed over the same period. We further explored the potential effect modification role of individual level education and simultaneous changes in the density of fruit and vegetable stores, chain convenience stores and supermarkets.

## 2 Methods

### 2.1 Setting

This study was conducted as part of the SALURBAL (SALud URBana en América Latina) Study (Diez Roux et al., 2019; Quistberg et al., 2018) that has compiled data on cities (defined as agglomerations of administrative units that share a common urban built-up extent) with a population of 100,000 or more in Latin America. We restricted our analysis to the 53 cities of Mexico with person-level data on diagnosed diabetes. This included 149 neighborhoods defined as *Áreas Geoestadísticas Básicas* (AGEB, the equivalent of a US census tract). An urban AGEB is made up by a group of blocks (1–50) and delimited by streets or avenues (Instituto Nacional de Geografía y Estadística, 2010b).

### 2.2 Person-level data

Person-level data were obtained from the National Health and Nutrition Survey of 2016 (*ENSANUT*) which is a cross-sectional population-based household survey carried out to collect information on nutrition, health and health related services and interventions. The design of the sample included stratification and multistage sampling to ensure representativeness at the national, regional and urban/rural levels (Romero-Martinez et al., 2017). We used data from 8824 adults aged ≥20 years that completed the survey's health, socioeconomic and demographic modules. We then selected adults living in urban areas, defined here as cities with ≥100,000 inhabitants (n = 3333). Rural areas were excluded because commercial food establishments are not well-represented in the database (rural food environments have much higher density of food cultivation and informal food establishments). Observations with incomplete information on the variables of interest (n = 306, 9%), and gestational diabetes cases (n = 6) were excluded (see appendix Table S1 for basic characteristics of complete and incomplete observations). Subsequently, we excluded individuals diagnosed with diabetes before 2010 (n = 213) because the exposure variable is a measure of change in the food environment from 2010 to 2016 as described below. The final sample consisted of 2808 men and women aged 20 years-old and older living in 149 neighbourhoods of 53 cities in Mexico. See appendix Fig. 2 for detailed sample flowchart. Survey participants were geolocated to the AGEB level.

### 2.3 Retail food environment data

Retail food environment data were obtained from the National Statistic Directory of Economic Units of 2010and 2016 (acronym in Spanish *DENUE*) which is an inventory of five million non-itinerant economic units related to manufacturing, commerce and services, their main economic activity and location (Instituto Nacional de Geografía y Estadística, 2018). The information for this directory is based on the National Economic Censuses which occur every five years (2009, 2014, 2019 being the latest), and updated on a yearly basis using economic surveys and field work coordinated by the National Institute of Geography and Statistics (United Nations Economic Commission for Europe, 2011).

We classified food stores as follows: fruit and vegetable stores, supermarkets, chain convenience stores, small food retail and fresh food retail (see appendix Table S2 for details). The North American Industrial Classification (NAICS) (United States Census Bureau, 2017) was used to identify fruit and vegetable stores, supermarkets, small food stores and fresh food stores. The NAICS was developed jointly by the US, Canada and Mexico economic and statistics offices to allow comparability in business statistics (United States Census Bureau, 2017). Chain convenience stores were searched by name since NAICS codes do not distinguish this type of store format. Chain convenience stores typically are less than 500 m^2^ in size, open ≥18 h a day for 365 days a year and sell mainly processed and ultra-processed food products and beverages. Six companies control 90% of the market and were included in the chain convenience store classification: OXXO, 7-Eleven, Extra, Circle K, Bodega Aurrera Express and Chedraui Supercito (America Retail, 2018).

We calculated the density of food stores within each neighborhood by dividing the number of establishments by the area in km^2^. We then calculated the change in the density of each store type from 2010 to 2016 and created a categorical variable for change (no change, decrease in density, increase in density). Person-level data were linked to neighborhood level data using a unique AGEB identifier. Only neighborhoods with person-level data were kept for analyses, therefore neighborhoods included are a sample of the total number of AGEBs in cities.

### 2.4 Outcome variable

The main outcome of this study was diabetes cases diagnosed on or after 2010 (yes/no), with cases defined as individuals that responded having been diagnosed with diabetes or 'high blood sugar' by a doctor from 2010 to 2016. The year of diagnosis was self-reported by the study participant. Gestational diabetes cases were excluded from the analyses since gestational diabetes is a temporary condition which usually resolves after birth.

### 2.5 Person-level covariates

We used data on sex (male or female), age (in years, continuous), educational attainment (incomplete high school, completed high school or above) and a household wealth index (in tertiles) (Howe et al., 2008). The wealth index was constructed using principal components analysis from household assets and characteristics –ownership of refrigerator, vehicle, computer, pay TV, internet connection and washing machine, household floor material, number of rooms, number of lightbulbs and water source. Household wealth indexes are commonly used in low- and middle-income countries as a proxy for consumption expenditure (Howe et al., 2012). Asset ownership and household quality characteristics are likely based at least partially on economic wealth and unlikely to change in response to short-term economic shocks. A detailed description of the methodology used to construct the index can be found elsewhere (Pérez-Ferrer et al., 2018).

### 2.6 Area-level covariates

We selected a number of neighborhood-level covariates that may be related to both changes in density of fruit and vegetable stores, chain convenience stores and supermarkets and diagnosed diabetes. These included: change in density of all small food retail, change in density of fresh food retail (calculated as described above for fruits and vegetables), density of sports and recreational facilities (DENUE 2016), marginalization index, proportion of the population without health insurance whether public or private and population density (population/km^2^). The last two variables were derived from the 2010 census and the State and Municipal Data Base System (Instituto Nacional de Geografía y Estadística, 2010a, c). The marginalization index is an area-level deprivation measure calculated by the National Population Council based on the 2010 census that incorporates 10 indicators from four dimensions: education, health, housing and assets (Consejo Nacional de Población, 2010). We chose these covariates because we hypothesize they have an impact on diabetes, and will be correlated with our main exposure (changes in the food environment).

### 2.7 Statistical analyses

The overarching goals of this analysis were to explore the changes in the composition of the retail food environment in Mexico and to study the association between these changes and diabetes cases diagnosed from 2010 to 2016. First, we described the neighborhood food environment for the period 2010–2016. We present the absolute number of each store type by year and its percent change. Second, we studied the association between the change in neighborhood food environment and person-level diagnosed diabetes. We used two-level logistic regression with random intercepts for neighborhood to account for correlations in diabetes response among persons living within the same neighborhood.

The outcome variable was recently (2010–2016) diagnosed diabetes and the exposure variables were change in fruit and vegetable store density, change in supermarket density, and change in chain convenience store density at neighborhood level. The focus was on these three store formats as exposures because 1. Fruit and vegetable stores unambiguously sell ‘healthy food’, as opposed to other small stores which sell both healthy and unhealthy foods and 2. We were interested in investigating the effect of the recent changes in the food environment which have been driven by the growth of chain convenience stores and supermarkets. Exposure variables were entered together into the model. The model was adjusted progressively to first understand how much of the variability in diabetes was accounted for by person characteristics and then by area-level factors (Yamada et al., 2020). Next we explored whether the association between the change in food store density and diabetes was modified by person-level educational attainment. We also explored whether change in convenience store and supermarket density modified the association between change in fruit and vegetable stores and diabetes. Interaction terms were added to separate (one for each interaction) fully adjusted models.

We performed three sensitivity analyses. First, we adjusted the model for the baseline (2010) density of each store type to account for the absolute number of stores at the beginning of the study period and isolate the effect of change over time. Second, we constructed the food store change variables in a different way; change from 2010 to 2016 for each type of store was centered and standardized by subtracting the mean for each observation and dividing by the standard deviation (SD). For fruit and vegetable stores, we further categorized the standardized variable into seven categories: 1. Decline in density of more than two SD; 2. Decline in density of between one and two SD; 3. decline of between zero and one SD; 4. no change in density, 5. increase of between zero and one SD; 6. Increase of between one and two SD; 7. Increase of more than two SD. There was not enough variation in the change in convenience store and supermarket density to create a similar variable. We ran the models again with these new variables. In the third and final sensitivity analysis we introduced a lag between exposure and outcome to explore if the estimated associations could reflect some underlying causal mechanism. We constructed variables for change in the food environment (as described for the main analyses) from 2010 to 2014 and kept only cases of diabetes diagnosed on or after 2015 (N = 74). Then we used two-level logistic regression with random intercepts for neighborhood to investigate the association between them in fully adjusted models.

All analyses were conducted in STATA 14 (StataCorp, College Station, TX). We present results from analyses that do not use survey weights due to evidence indicating the complexity of using them when fitting multilevel models and minimal differences from scaled weighted and unweighted estimates (Yamada et al., 2020).

## 3 Results

Table 1 shows the characteristics of the sample in 2016. The mean age was 45.4 years old, 73.8% had less than complete high school education. One hundred and eighty-five individuals or 6.6% of the sample were diagnosed with diabetes between 2010 and 2016.

**Table 1:** Descriptive characteristics of adults (ENSANUT, 2016) and neighborhoods (N = 149) in the analytic sample.

Variables	N (%)	Mean (SD)
Individual level N = 2808		
Sex, %		
Male	909 (32.4)	
Female	1899 (67.6)	
**Age**		45.4 (16.6)
**Education, %**		
Incomplete high school or less	2071 (73.8)	
Complete high school or more	737 (26.3)	
**Wealth, %**		
Tertile 1 (poorest)	512 (18.2)	
Tertile 2	841 (30.0)	
Tertile 3 (richest)	1455 (51.8)	
**Diabetes (new cases 2010–2016), %**	185 (6.6)	
**Neighborhood level N** = **149**		
Proportion without health insurance (2010)		34.3 (11.5)
Availability of sports and recreational facilities (2016), % (SD)		49.0 (50.2)
Marginalization index (2010)		−0.2 (0.7)
Population density per km2 (2010)		11543.2 (8081.7)

The first three columns of Table 2 describe the total number of food stores by type for all 149 neighborhoods included in this study in 2010 and 2016. Overall, there was a small increase (3%) in the total number of food stores over time. While small food retail stores were far more numerous than chain convenience stores and supermarkets, their number declined slightly over time (1% decline from 2010 to 2016). We found 496 and 516 fruit and vegetable stores in 2010 and 2016, respectively, representing a 4% increase over time. In comparison, we found 40 and 71 chain convenience stores and 5 and 9 supermarkets in 2010 and 2016, respectively, representing a 77.5% and 80% increase over time. In summary, we found that the share of convenience stores and supermarkets, relative to fruit and vegetable stores, increased over time: in 2010, there were 12 and 99 fruit and vegetable stores per convenience store and supermarket, respectively, but these numbers declined by around 40% to 7 and 57 fruit and vegetable stores to convenience stores and supermarkets, respectively.

**Table 2:** Total number of food stores in 149 neighborhoods included in the sample, and proportion of neighborhoods with one store or more in 2010 and 2016.

	Total number of stores (%)			N (%) AGEBs with ≥1 store		
	2010	2016	Change	2010	2016	Change
Total stores selling food	5304 (100.0)	5465 (100.0)	161 (3.0)	149 (100)	149 (100)	0
-Small food retail	4101 (77.3)	4061 (74.3)	−40 (−1.0)	149 (100)	149 (100)	0
--Fresh food retail	662 (12.5)	808 (14.8)	146 (22.1)	122 (81.9)	123 (82.6)	1 (0.8)
---Fruit and vegetable stores	496 (9.4)	516 (9.4)	20 (4.0)	101 (67.8)	117 (78.5)	16 (15.8)
Chain convenience stores	40 (0.8)	71 (1.3)	31 (77.5)	31 (20.8)	51 (34.2)	29 (64.5)
Supermarkets	5 (0.1)	9 (0.2)	4 (80.0)	4 (2.7)	8 (5.4)	4 (100.0)
Ratio FV:convenience	12.4	7.3	−41.4%			
Ratio FV:supermarket	99.2	57.3	−42.2%			
Ratio FV:convenience + supermarket	11.0	6.5	−41.5%			

FV: Fruit and vegetable store.

**Fig. 1:**
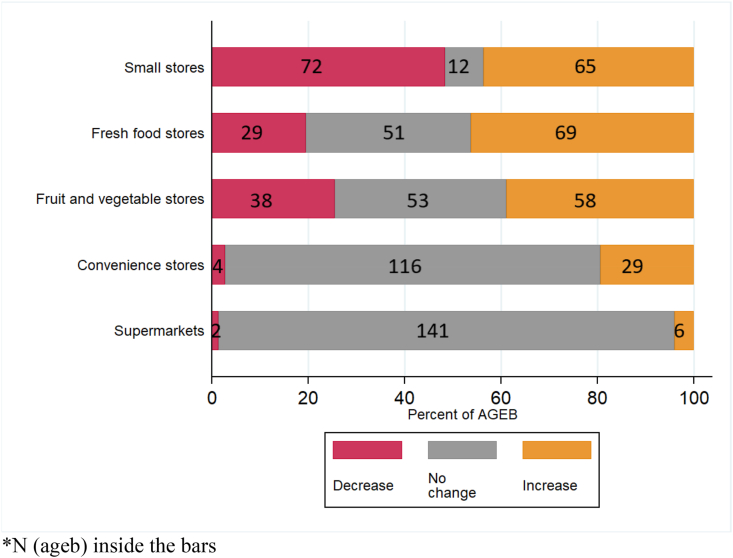
Change in the density of food stores at AGEB level, 2010–2016.

All neighborhoods included in the sample (149) had at least one small food retail store in 2010 and 2016 (Table 2, last three columns). Sixty-eight percent had at least one fruit and vegetable store in 2010 increasing to 78.5% in 2016. Chain convenience stores were present in 31 out of 149 neighborhoods (20.8%) in 2010 increasing to 51 of 149 (34.2%) in 2016, while the number of neighborhoods with a supermarket in this sample doubled from 4 to 8. Fig. 1 shows that at the neighborhood level, the density of chain convenience stores and supermarkets did not change in 77.9% and 94.6% of AGEBs respectively. Chain convenience stores and supermarkets increased in 19.5% and 4.0% of AGEBs. There was more change in the density of fruit and vegetable stores at neighborhood level over the period 2010–2016. In 25.5% of AGEBs, the density of fruit and vegetable store declined while in 35.6% the number of these types of stores did not change, and in 38.9% the density increased.

Table 3 shows the association between the change in the neighborhood density of fruit and vegetable stores, chain convenience stores and large supermarkets and recently (2010–2016) diagnosed diabetes. The adjusted models suggest that adults living in neighborhoods that saw a decline in fruit and vegetable store density experienced higher odds of diabetes compared to adults who lived in neighborhoods where fruit and vegetable store density remained stable (OR 1.64 95%CI 1.06, 2.53). The association was robust to adjustments for confounders at the individual and neighborhood levels. Changes in the density of convenience stores and supermarkets were not associated with diabetes.

**Table 3:** Association between change in food store density (2010–2016) at neighborhood level and odds of diabetes in 2016.

Density change	Sample size	Diabetes cases	Model 1	Model 2	Model 3
**Fruit and vegetable shops, change**					
1 (decline)	647	57	**1.66 (1.13,2.43)**	**1.61 (1.09,2.38)**	**1.64 (1.06,2.53)**
2 (no change)	1041	57	1.00	1.00	1.00
3 (increase)	1120	71	1.16 (0.81,1.67)	1.21 (0.84,1.75)	1.26 (0.86,1.87)
**Convenience stores, change**					
1 (no change)	2256	147	1.00	1.00	1.00
2 (increase)	552	38	1.02 (0.85,1.23)	1.01 (0.83,1.22)	1.04 (0.85,1.27)
**Supermarkets, change**					
1 (no change)	2688	178	1.00	1.00	1.00
2 (increase)	120	7	0.96 (0.65,1.43)	1.04 (0.69,1.55)	1.00 (0.66,1.53)

Model 1: No adjustments; Model 2: adjusted for sex, age, wealth tertile and education level; Model 3: Model 2 + population density, marginalization index, change in small food retail, change in fresh food, presence of sports facilities and proportion of the population without health insurance (public or private). Tertiles of convenience store and supermarket change at ageb level were collapsed into two categories because of variable distribution (predominance of zero values).

**Fig. 4:**
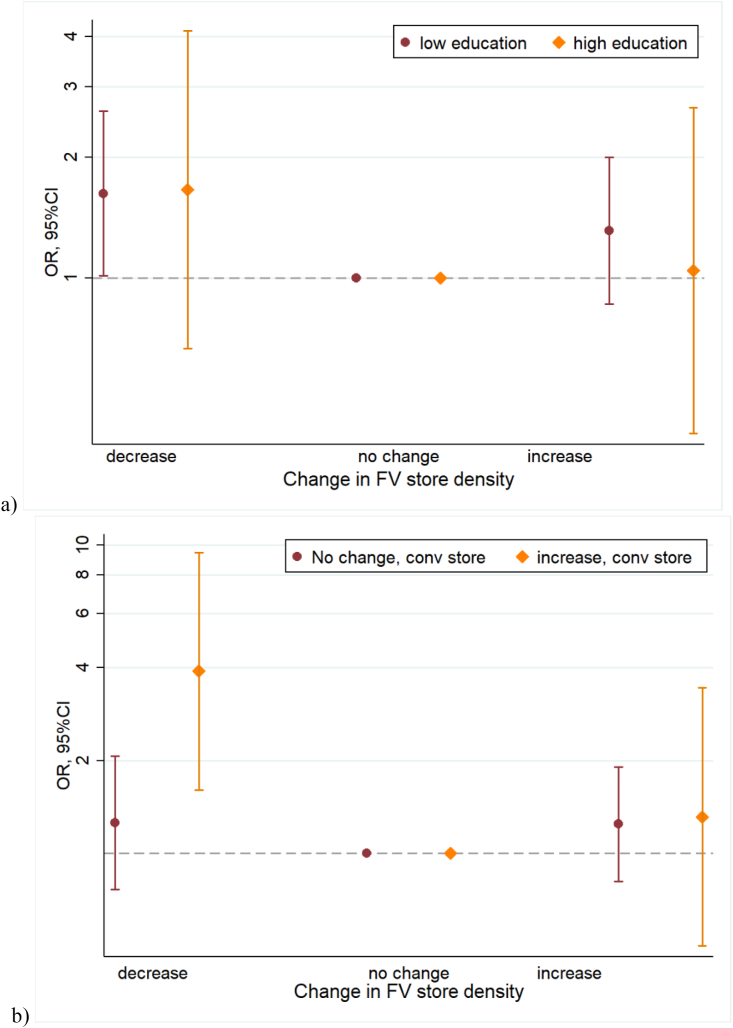
Association between change in the density of fruit and vegetable stores and odds of diabetes in 2016 by a) education level and b) change in chain convenience store density.

Fig. 4a shows that the association between changes in fruit and vegetable store density and recently diagnosed diabetes was similar for those with higher and lower education (p for interaction = 0.27). Fig. 4b shows that the association between a decline in fruit and vegetable store density and recently diagnosed diabetes was only significant among individuals who were living in areas where there was a simultaneous increase in convenience stores (OR 3.90 95% CI 1.61,9.48) and not in areas with no change in convenience stores (OR 1.25 95%CI 0.76, 2.06; interaction p = 0.025). The association between fruit and vegetable store change and diabetes was not modified by change in supermarket density (Supplementary Fig. S5).

### 3.1 Sensitivity analyses

Supplementary table S3 shows that adjusting for baseline density of all store types did not change the results. Supplementary Table S4 shows the results of the sensitivity analysis where standardized change variables were used as the exposure. Results were analogous to the main findings, with declines of up to 1 standard deviations in fruit and vegetable stores associated with increased odds of diabetes (OR 1.80 95% CI 1.15, 2.81). Larger reductions in fruit and vegetable store density were not associated with diabetes, but we had very few neighborhoods and individuals in these categories. Consistent with the main findings, an increase of 1 standard deviation in chain convenience stores and supermarkets was not associated with the prevalence of diabetes.

Table S5 shows the association between change in the food environment from 2010 to 2014 and diabetes cases diagnosed on or after 2015. The results support the main findings; a decline in the density of fruit and vegetable stores was associated with increased odds of diabetes (OR 2.03 95%CI 1.14, 3.63) and there was no association between changes in convenience stores/supermarkets and diabetes.

## 4 Discussion

There are two key findings in this study of retail food environments and diabetes in Mexican urban areas: (1) small store formats still predominate in the food environment of Mexico, however there is evidence of a fast increase in chain convenience stores and supermarkets; (2) people living in neighborhoods with a decline in the density of fruit and vegetable stores and a simultaneous increase in chain convenience stores had higher odds of diabetes.

Previous studies have documented the growth in supermarkets and chain convenience stores in Mexico associated with the North American Free Trade Agreement (NAFTA) signed in the 1990s (Hawkes, 2006). The number of chain supermarkets and convenience stores increased from less than 700 in 1997 to 5729 in 2004, i.e. over 100% increase per year (Hawkes, 2006). Our study documents the continuation of this trend although the rate of increase seems to have slowed down (approximately 13% per year increase in urban neighborhoods). This study also shows that although the relative increase in supermarkets and chain convenience stores is large, traditional food retail channels, such as *tiendas de abarrotes* and fresh food stores, continue being the predominant type of store in Mexico, with 67 small food retailers per supermarket or chain convenience store. Small food stores are generally family owned and vary significantly in their stock. Some sell both healthy and unhealthy foods while others sell mainly ultra-processed foods. These stores are key to the distribution of ultra-processed foods in Mexico, accounting for an important proportion of the sales of transnational food companies including sugar sweetened beverages, baked goods, snacks, beer and dairy (Pallares, 2013). Small stores face growing competition from new chain convenience stores and supermarkets however, they have been resilient, and their number have declined only slightly (1% decline 2010–2016). Fresh food stores including butchers, poultry shops, fishmongers and other specialized shops increased over the study period, regardless of competition from new supermarkets.

Findings from this study on the association between the density of fruit and vegetable stores and diabetes are consistent with other Latin American studies which have shown that a higher density of these types of stores are associated with favorable nutrition outcomes (Duran et al., 2016; Jaime et al., 2011; Molina et al., 2017; Pessoa et al., 2015). This study investigated this association further by looking at it in the context of other changes in the food environment and found that fruit and vegetable stores were only significantly associated with diabetes in neighborhoods that also saw an increase in chain convenience stores, not in areas with no change in convenience stores. This is consistent with the idea that both healthy and unhealthy food stores may be associated with diabetes. Specifically, neighborhoods with a decline in fruit and vegetable stores and an increase in convenience stores face a doubly-negative change in the food environment, which may have an impact on diet and diet related diseases such as diabetes. If the protective role of fruit and vegetable stores is confirmed with longitudinal studies and/or health experiments, findings can be used to design place-based policies at the city and neighborhood level. Currently, policies targeting the retail food environment in Mexico are lacking (Nieto et al., 2019). There are examples from other countries where there are local policies that support fruit and vegetable stores and guarantee supply of healthy food at affordable prices (Lopes et al., 2017).

We did not observe an independent association between changes in the density of chain convenience stores and supermarkets with diabetes. These findings are consistent with studies from the USA that have found null or weak association between grocery stores and supermarkets with diabetes (Bilal et al., 2018a). The number of chain convenience stores and supermarkets is still small relative to independent small food stores but their relative share continues to increase. It is possible that we were not able to detect an effect from changes in convenience stores and supermarkets due to little variation observed at the neighborhood level. It is therefore important to continue monitoring their growth, potential changes in their stock, and potential impacts on health outcomes.

We acknowledge some limitations. First, we have food retail environment data from 2010 onwards, so we are only able to capture the last six years of supermarket and convenience store growth, missing part of the exponential growth that occurred in the 1990s and 2000s (Hawkes, 2006). Second, we relied on self-reported diagnosed diabetes. Self-reported diabetes is commonly used in surveillance surveys and has previously been found to be a valid measurement of prevalent diabetes (Schneider et al., 2012). The estimated prevalence of total diabetes (diagnosed + undiagnosed) in Mexico is 13.7% (Basto-Abreu et al., 2020). If diabetes is under-diagnosed among those with lower socio-economic levels (Basto-Abreu et al., 2020), then our results may be a conservative estimate of the association between increases in convenience stores/supermarket density and diabetes (based on the assumption that lower SES areas were exposed to greater increases in convenience stores and supermarkets (Duran et al., 2013)). Further, our interest was studying diabetes type 2 which is associated with modifiable factors such as diet. The survey question on diabetes diagnosis was not specific to this type of diabetes. However, the prevalence of type 1 diabetes is low (less than 0.5% in the United States, unknown for Mexico) and three fourths of cases are diagnosed in childhood and adolescence (Maahs et al., 2010). While it is still possible that the outcome variable may have some measurement error, by focusing this study on adults and eliminating diagnosis made before 2010, we likely eliminated most cases of type 1 diabetes. An additional point regarding the outcome variable is that there may be recall bias for the question on year of diagnosis. Potentially by focusing on more recent diagnoses, we may have limited the effect of this bias on our results. Third, we use cross-sectional individual level data therefore, temporality in the associations cannot be established. To account for some of this, we restricted the analytic sample to individuals diagnosed with diabetes on or after 2010 to ensure that diagnosis occurred at least at the same time as changes in the food environment. We also carried out the sensitivity analysis where we introduced a lag and found very similar results to the main analysis. Future studies using longitudinal data or natural experiments (Franco et al., 2013; Ghosh-Dastidar et al., 2017) are needed to better understand the causal pathways between the food environment and nutrition and health outcomes. In addition, due to the cross-sectional nature of the data we do not know whether survey respondents have moved during this time period, meaning they might have been exposed to other food environments over the 6-year period. Fourth, the economic census relies on economic activity data (NAICS code), but there may still be significant heterogeneity within types of stores, with some supermarkets selling healthier foods as compared to others (Cannuscio et al., 2013; Chrisinger et al., 2018), leading to potential measurement error. Consumer food environment studies with in-store audits are needed in Mexico in order to better classify stores and identify those that are more relevant to health and disease. Finally, this study used the neighborhood as the spatial unit of analysis for the food environment. This is a strength of this study since no previous studies in Mexico had analyzed the food environment at this level and because we were able to construct a more precise measure of exposure compared to aggregating information at the municipality level or higher. However, the analysis at the neighborhood level also presented limitations because there was limited variability in change over the period for a large number of neighborhoods. Further, people might not always shop close to home (Bilal et al., 2018a; Chrisinger et al., 2018), and the neighborhood level does not capture the effect of the food environment in a wider area of influence.

## 5 Conclusion

We found that, adults living in neighborhoods that saw a decline in fruit and vegetable store density and a simultaneous increase in chain convenience store density experienced higher odds of diabetes, compared to adults who lived in neighborhoods where fruit and vegetable and convenience stores did not change. Considering the complex interplay between store types, understanding the mechanisms and confirming the causal implications of these findings could inform policies that improve the quality of food environments in Latin American cities.

## Funding

This work is part of the *Salud Urbana en América Latina* (SALURBAL)/Urban Health in Latin America project funded by the 10.13039/100010269Wellcome Trust [205177/Z/16/Z]. More information about the project can be found at www.lacurbanhealth.org. The funder had no role in the design, analysis or write up of this article.

## Declaration of competing interest

None.

## References

[bib1] Basto-Abreu A., Barrientos-Gutiérrez T., Rojas-Martínez R., Aguilar-Salinas C.A., López-Olmedo N., De la Cruz-Góngora V., Rivera-Dommarco J., Shamah-Levy T., Romero-Martínez M., Barquera S., López-Ridaura R., Hernández-Ávila M., Villalpando S. (2020). [Prevalence of diabetes and poor glycemic control in Mexico: results from Ensanut 2016.]. Salud Publica Mex..

[bib2] Bilal U., Auchincloss A.H., Diez-Roux A.V. (2018). Neighborhood environments and diabetes risk and control. Curr. Diabetes Rep..

[bib3] Bilal U., Jones-Smith J., Diez J., Lawrence R.S., Celentano D.D., Franco M. (2018). Neighborhood social and economic change and retail food environment change in Madrid (Spain): The heart healthy hoods study. Health Place.

[bib4] Cannuscio C.C., Tappe K., Hillier A., Buttenheim A., Karpyn A., Glanz K. (2013). Urban food environments and residents' shopping behaviors. Am. J. Prev. Med..

[bib5] Cobb L.K., Appel L.J., Franco M., Jones-Smith J.C., Nur A., Anderson C.A. (2015). The relationship of the local food environment with obesity: A systematic review of methods, study quality, and results. Obesity.

[bib6] Chrisinger B.W., Kallan M.J., Whiteman E.D., Hillier A. (2018). Where do U.S. households purchase healthy foods? An analysis of food-at-home purchases across different types of retailers in a nationally representative dataset. Prev. Med..

[bib7] Christine P.J., Auchincloss A.H., Bertoni A.G., Carnethon M.R., Sanchez B.N., Moore K., Adar S.D., Horwich T.B., Watson K.E., Diez Roux A.V. (2015). Longitudinal associations between neighborhood physical and social environments and incident Type 2 diabetes mellitus: The multi-ethnic study of atherosclerosis (MESA). JAMA Intern Med.

[bib8] Diez Roux A.V., Slesinski S.C., Alazraqui M., Caiaffa W.T., Frenz P., Jordán Fuchs R., Miranda J.J., Rodriguez D.A., Dueñas O.L.S., Siri J., Vergara A.V. (2019). A novel international partnership for actionable evidence on urban health in Latin America: LAC-urban health and SALURBAL. Global Challenges.

[bib9] Duran A.C., de Almeida S.L., Latorre M., Jaime P.C. (2016). The role of the local retail food environment in fruit, vegetable and sugar-sweetened beverage consumption in Brazil. Publ. Health Nutr..

[bib10] Duran A.C., Diez Roux A.V., Latorre M., Jaime P.C. (2013). Neighborhood socioeconomic characteristics and differences in the availability of healthy food stores and restaurants in Sao Paulo, Brazil. Health Place.

[bib11] Franco M., Bilal U., Ordunez P., Benet M., Morejon A., Caballero B., Kennelly J.F., Cooper R.S. (2013). Population-wide weight loss and regain in relation to diabetes burden and cardiovascular mortality in Cuba 1980-2010: repeated cross sectional surveys and ecological comparison of secular trends. Bmj.

[bib12] Gebreab S.Y., Hickson D.A., Sims M., Wyatt S.B., Davis S.K., Correa A., Diez-Roux A.V. (2017). Neighborhood social and physical environments and type 2 diabetes mellitus in African Americans: The Jackson Heart Study. Health Place.

[bib13] Ghosh-Dastidar M., Hunter G., Collins R.L., Zenk S.N., Cummins S., Beckman R., Nugroho A.K., Sloan J.C., Wagner L., Dubowitz T. (2017). Does opening a supermarket in a food desert change the food environment?. Health Place.

[bib14] Glanz K., Sallis J.F., Saelens B.E., Frank L.D. (2005). Healthy nutrition environments: concepts and measures. Am. J. Health Promot..

[bib15] Hawkes C. (2006). Uneven dietary development: linking the policies and processes of globalization with the nutrition transition, obesity and diet-related chronic diseases. Glob. Health.

[bib16] Hinnig P.F., Monteiro J.S., de Assis M.A.A., Levy R.B., Peres M.A., Perazi F.M., Porporatti A.L., Canto G.L. (2018). Dietary patterns of children and adolescents from high, medium and low human development countries and associated socioeconomic factors: a systematic review. Nutrients.

[bib17] Howe L.D., Galobardes B., Matijasevich A., Gordon D., Johnston D., Onwujekwe O., Patel R., Webb E.A., Lawlor D.A., Hargreaves J.R. (2012). Measuring socio-economic position for epidemiological studies in low- and middle-income countries: a methods of measurement in epidemiology paper. Int. J. Epidemiol..

[bib18] Imamura F., O'Connor L., Ye Z., Mursu J., Hayashino Y., Bhupathiraju S.N., Forouhi N.G. (2015). Consumption of sugar sweetened beverages, artificially sweetened beverages, and fruit juice and incidence of type 2 diabetes: systematic review, meta-analysis, and estimation of population attributable fraction. Bmj.

[bib19] Jaime P.C., Duran A.C., Sarti F.M., Lock K. (2011). Investigating environmental determinants of diet, physical activity, and overweight among adults in sao paulo, Brazil. Journal of Urban Health-Bulletin of the New York Academy of Medicine.

[bib20] Lopes A.C.S., Menezes M.C.d., Araújo M.L.d. (2017). O ambiente alimentar e o acesso a frutas e hortaliças: Uma metrópole em perspectiva. Saúde e Sociedade.

[bib21] Maahs D.M., West N.A., Lawrence J.M., Mayer-Davis E.J. (2010). Epidemiology of type 1 diabetes. Endocrinol Metab. Clin. N. Am..

[bib22] Mendes L.L., Nogueira H., Padez C., Ferrao M., Velasquez-Melendez G. (2013). Individual and environmental factors associated for overweight in urban population of Brazil. BMC Publ. Health.

[bib23] Mezuk B., Li X., Cederin K., Rice K., Sundquist J., Sundquist K. (2016). Beyond access: characteristics of the food environment and risk of diabetes. Am. J. Epidemiol..

[bib24] Molina M., Servan-Mori E., Quezada A.D., Colchero M.A. (2017). Is there a link between availability of food and beverage establishments and BMI in Mexican adults?. Publ. Health Nutr..

[bib25] Nieto C., Rodriguez E., Sanchez-Bazan K., Tolentino-Mayo L., Carriedo-Lutzenkirchen A., Vandevijvere S., Barquera S. (2019). The INFORMAS healthy food environment policy index (Food-EPI) in Mexico: An assessment of implementation gaps and priority recommendations. Obes. Rev..

[bib26] Perez-Ferrer C., Auchincloss A.H., de Menezes M.C., Kroker-Lobos M.F., Cardoso L.O., Barrientos-Gutierrez T. (2019). The food environment in Latin America: a systematic review with a focus on environments relevant to obesity and related chronic diseases. Publ. Health Nutr..

[bib27] Pérez-Ferrer C., McMunn A., Zaninotto P., Brunner E.J. (2018). The nutrition transition in Mexico 1988-2016: the role of wealth in the social patterning of obesity by education. Publ. Health Nutr..

[bib28] Pessoa M.C., Mendes L.L., Gomes C.S., Martins P.A., Velasquez-Melendez G. (2015). Food environment and fruit and vegetable intake in a urban population: A multilevel analysis. BMC Publ. Health.

[bib29] Popkin B.M. (2015). Nutrition Transition and the global diabetes epidemic. Curr. Diabetes Rep..

[bib30] Qian F., Liu G., Hu F.B., Bhupathiraju S.N., Sun Q. (2019). Association between plant-based dietary patterns and risk of Type 2 diabetes: A systematic review and meta-analysis. JAMA Intern Med.

[bib31] Quistberg D.A., Diez Roux A.V., Bilal U., Moore K., Ortigoza A., Rodriguez D.A., Sarmiento O.L., Frenz P., Friche A.A., Caiaffa W.T., Vives A., Miranda J.J. (2018). Building a Data Platform for Cross-Country Urban Health Studies: the SALURBAL Study.

[bib32] Rojas-Martinez R., Basto-Abreu A., Aguilar-Salinas C.A., Zarate-Rojas E., Villalpando S., Barrientos-Gutierrez T. (2018). [Prevalence of previously diagnosed diabetes mellitus in Mexico.]. Salud Publica Mex..

[bib33] Romero-Martinez M., Shamah-Levy T., Cuevas-Nasu L., Gomez-Humaran I.M., Gaona-Pineda E.B., Gomez-Acosta L.M., Rivera-Dommarco J.A., Hernandez-Avila M. (2017). [Methodological design of the national health and nutrition survey 2016]. Salud Publica Mex..

[bib34] Schneider A.L.C., Pankow J.S., Heiss G., Selvin E. (2012). Validity and reliability of self-reported diabetes in the atherosclerosis risk in communities study. Am. J. Epidemiol..

[bib35] Schwingshackl L., Hoffmann G., Lampousi A.M., Knuppel S., Iqbal K., Schwedhelm C., Bechthold A., Schlesinger S., Boeing H. (2017). Food groups and risk of type 2 diabetes mellitus: a systematic review and meta-analysis of prospective studies. Eur. J. Epidemiol..

[bib36] USDA Foreign Agricultural Service (2016). Mexico Retail Foods. 2016 Annual Report.

[bib37] World Health Organization (2011). Global Status Report on Noncommunicable Diseases 2010.

[bib38] Yamada G., Jones-Smith J.C., Castillo-Salgado C., Moulton L.H. (2020). Differences in magnitude and rates of change in BMI distributions by socioeconomic and geographic factors in Mexico, Colombia, and Peru, 2005-2010. Eur. J. Clin. Nutr..

