# Supplementary material for: Longitudinal changes in the retail food environment in Mexico and their association with diabetes
Source: Health Place. 2020 Nov;66:102461. doi: 10.1016/j.healthplace.2020.102461 (PMC7705211; doi:10.1016/j.healthplace.2020.102461)
Supplement: Multimedia component 1 [file mmc1.docx]

**Longitudinal Changes in the Retail Food Environment in Mexico and their Association with Diabetes**

Authors:

Carolina Pérez-Ferrer

Amy H. Auchincloss

Tonatiuh Barrientos-Gutierrez

M. Arantxa Colchero

Leticia de Oliveira Cardoso

Mariana Carvalho de Menezes

Usama Bilal

**Supplemental files**

**File contents**

[Figure S1. Causal framework underlying the research question 2](#_Toc44501232)

[Figure S2. Flowchart for sample selection 3](#_Toc44501233)

[Table S1. Comparison of demographic characteristics of complete sample and sample with incomplete observations 4](#_Toc44501234)

[Table S2. Food store classification 5](#_Toc44501235)

[Table S3. Association between change in density of food stores and diabetes, adjusted for baseline density 6](#_Toc44501236)

[Table S4. Association of change measured in terms of standard deviations and diabetes 7](#_Toc44501237)

[Table S5. Association between change in the food environment from 2010 to 2014 and diabetes cases diagnosed on or after 2015 7](#_Toc44501238)

[Figure S3. Association between change in density of convenience stores and odds of diabetes by education level 9](#_Toc44501239)

[Figure S4. Association between change in supermarket density and odds of diabetes by education level 10](#_Toc44501240)

[Figure S5. Association between change in fruit and vegetable store density and odds of diabetes by change in supermarket density. 11](#_Toc44501241)

[References for suplemental material 12](#_Toc44501242)

Figure S1. Causal framework underlying the research question


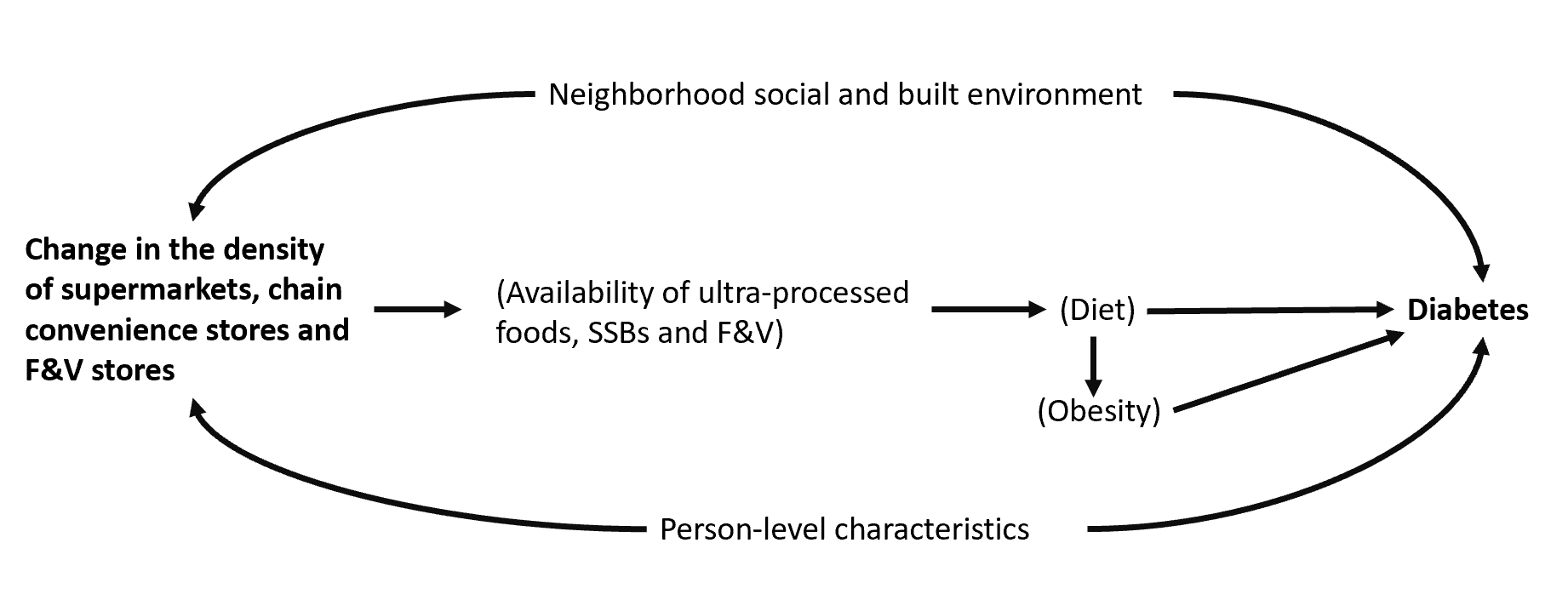


*In parentheses are variables hypothesized to be on the pathway but that are not included in this study.

Figure S2. Flowchart for sample selection


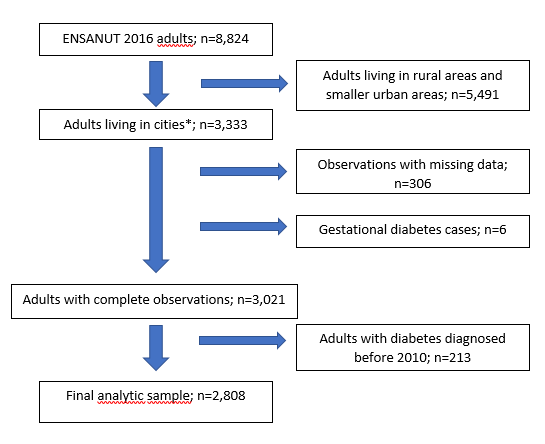


*Cities were defined as agglomerations of administrative units that share a common urban built-up extent with a population of 100,000 or more as per the SALURBAL Project protocol ^(1)^

Table S1. Comparison of demographic characteristics of complete sample and sample with incomplete observations

|  | **Complete sample** | **Incomplete observations** | **p for difference** |
| --- | --- | --- | --- |
| N | 3,027 | 306 |  |
| Sex, proportion |  |  |  |
| Male | 46.3 | 54.6 |  |
| Female | 53.7 | 45.4 | 0.207 |
| Education level, proportion |  |  |  |
| Education low | 65.9 | 47.6 |  |
| Education high | 34.1 | 52.4 | 0.061 |
| Wealth index, proportion |  |  |  |
| Poorest | 13.1 | 7.2 | 0.032 |
| Middle | 26.7 | 14.6 | 0.002 |
| Richest | 60.2 | 78.1 | 0.001 |
| Age, mean | 42.0 | 39.2 | 0.070 |

Potential implications for study findings: Individuals with incomplete observations were richer and tended to be more educated, more male and younger. We could therefore assume that those individuals had a lower prevalence of diabetes and lived in areas with more favorable changes in the food environment (chain convenience stores have increased more rapidly in poorer neighborhoods). If this assumption were true, then diagnosed diabetes prevalence may be slightly overestimated and the association between the food environment and diabetes may be conservative.

Table S2. Food store classification

| Food store type | NAICS code |
| --- | --- |
| Fruit and vegetable stores | 461130 |
| Supermarkets | 462111 minus ‘chain convenience stores’ (see below) |
| Small food retail | 461110 (small grocery stores) + 461213 (non-alcoholic beverage stores) + 462112 (minimarkets) minus ‘chain convenience stores’ |
| Fresh food retail | 46112* (meat, poultry & fish shops) + 461150 (dairy shops) + 461140 (stores selling grains and seeds) |
| Chain convenience stores | Searched by name because NAICS does not identified them as a distinct store format. Names searched: OXXO, 7-Eleven, Extra, Circle K, Bodega Aurrera Express and Chedraui Supercito |

Table S3. Association between change in density of food stores and diabetes, adjusted for baseline density

| **Density change** | **Sample size** | **Diabetes cases** | **Model 4** |
| --- | --- | --- | --- |
| **Fruit and vegetable shops, change** | |  |  |
| 1 (decline) | 647 | 57 | **1.70 (1.07,2.70)** |
| 2 (no change) | 1041 | 57 | 1.00 |
| 3 (increase) | 1120 | 71 | 1.19 (0.80,1.78) |
| **Convenience stores, change** | |  |  |
| 1 (no change) | 2256 | 147 | 1.00 |
| 2 (increase) | 552 | 38 | 1.01 (0.82,1.24) |
| **Supermarkets, change** | |  |  |
| 1 (no change) | 2688 | 178 | 1.00 |
| 2 (increase) | 120 | 7 | 0.97 (0.64,1.49) |

Model 1: No adjustments; Model 2: adjusted for sex, age, wealth tertile and education level; Model 3: Model 2 + population density, marginalization index, change in small food retail, change in fresh food, presence of sports facilities and proportion of the population without health insurance (public or private). Model 4: adjusted for sex, age, wealth tertile and education level, population density, marginalization index, change in small food retail, change in fresh food, presence of sports facilities and proportion of the population without health insurance (public or private) **and baseline (2010) density of fruit and vegetable stores, convenience stores and supermarkets.**

Table S4. Association of change measured in terms of standard deviations and diabetes

| Change in FV stores | **Neighborhood N** | **No diabetes** | **Diabetes** | **Model 1, OR(95%CI)** | **Model 2, OR(95%CI)** | **Model 3, OR(95%CI)** |
| --- | --- | --- | --- | --- | --- | --- |
| -Decline of more than 2SD | 6 | 78 | 5 | 1.15 (0.45,2.96) | 1.01 (0.38,2.66) | 1.12 (0.39,3.24) |
| -Decline of between 1 and 2 SD | 7 | 126 | 11 | 1.45 (0.74,2.86) | 1.49 (0.75,2.98) | 1.19 (0.55,2.55) |
| -Decline of between 0 and 1SD | 25 | 386 | 41 | **1.89 (1.23,2.89)** | **1.88 (1.21,2.91)** | **1.91 (1.19,3.07)** |
| -0, no change | 53 | 984 | 57 | 1.00 (0.00,0.00) | 1.00 (0.00,0.00) | 1.00 (0.00,0.00) |
| -Increase of between 0 and 1 SD | 50 | 889 | 63 | 1.25 (0.86,1.82) | 1.32 (0.90,1.94) | 1.37 (0.92,2.04) |
| -Increase of between 1 and 2SD | 7 | 133 | 7 | 0.92 (0.41,2.07) | 0.99 (0.43,2.25) | 0.85 (0.35,2.07) |
| -Increase of more than 2SD | 1 | 27 | 1 | 0.66 (0.09,4.98) | 0.97 (0.13,7.41) | 0.81 (0.09,7.12) |
| Change in convenience, 1 SD | 149 | 2623 | 185 | 1.04 (0.90,1.21) | 1.00 (0.86,1.16) | 1.00 (0.85,1.17) |
| Change in supermarkets, 1SD | 149 | 2623 | 185 | 1.06 (0.90,1.26) | 1.08 (0.89,1.30) | 1.07 (0.88,1.29) |

Model 1: No adjustments; Model 2: adjusted for sex, age, wealth tertile and education level; Model 3: Model 2 + population density, marginalization index, change in small food retail, change in fresh food, presence of sports facilities and proportion of the population without health insurance (public or private).

Table S5. Association between change in the food environment from 2010 to 2014 and diabetes cases diagnosed on or after 2015

| **Density change** | **Sample size** | **Diabetes cases** | **Model 1** | **Model 2** | **Model 3** |
| --- | --- | --- | --- | --- | --- |
| **Fruit and vegetable shops, change** | |  |  |  |  |
| 1 (decline) | 618 | 26 | **2.03 (1.14,3.63)** | **1.98 (1.10,3.55)** | **2.26 (1.18,4.33)** |
| 2 (no change) | 1033 | 22 | 1.00 | 1.00 | 1.00 |
| 3 (increase) | 1046 | 26 | 1.20 (0.67,2.14) | 1.21 (0.67,2.17) | 1.26 (0.68,2.34) |
| **Convenience stores, change** | |  |  |  |  |
| 1 (no change) | 2207 | 59 | 1.00 | 1.00 | 1.00 |
| 2 (increase) | 59 | 15 | 1.06 (0.79,1.42) | 1.06 (0.79,1.42) | 1.12 (0.82,1.54) |
| **Supermarkets, change** | |  |  |  |  |
| 1 (no change) | 2510 | 70 | 1.00 | 1.00 | 1.00 |
| 2 (increase) | 117 | 4 | 1.17 (0.69,1.98) | 1.23 (0.72,2.09) | 1.09 (0.62,1.90) |
|  |  |  |  |  |  |

Model 1: No adjustments; Model 2: adjusted for sex, age, wealth tertile and education level; Model 3: Model 2 + population density, marginalization index, change in small food retail, change in fresh food, presence of sports facilities and proportion of the population without health insurance (public or private).

Figure S3. Association between change in density of convenience stores and odds of diabetes by education level
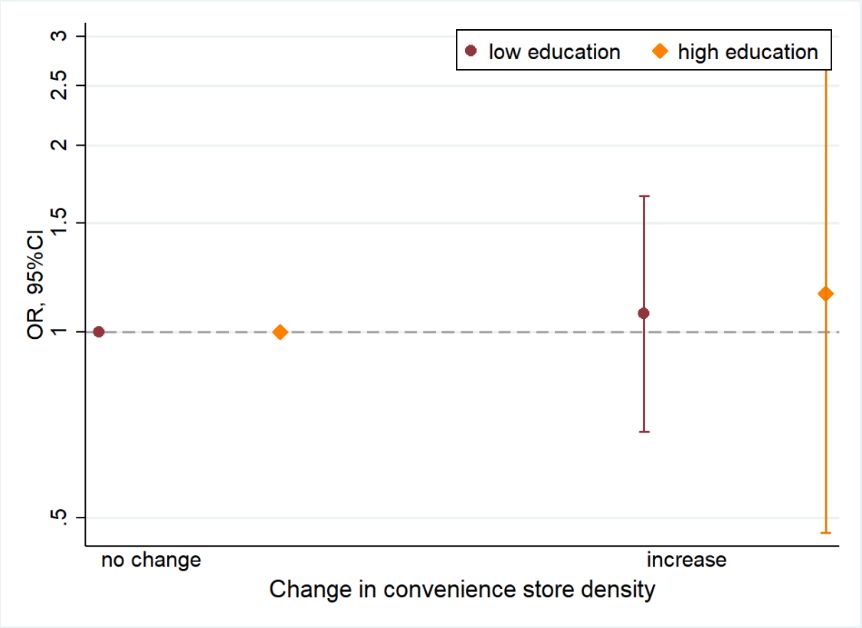


Interation p=0.6374

Figure S4. Association between change in supermarket density and odds of diabetes by education level


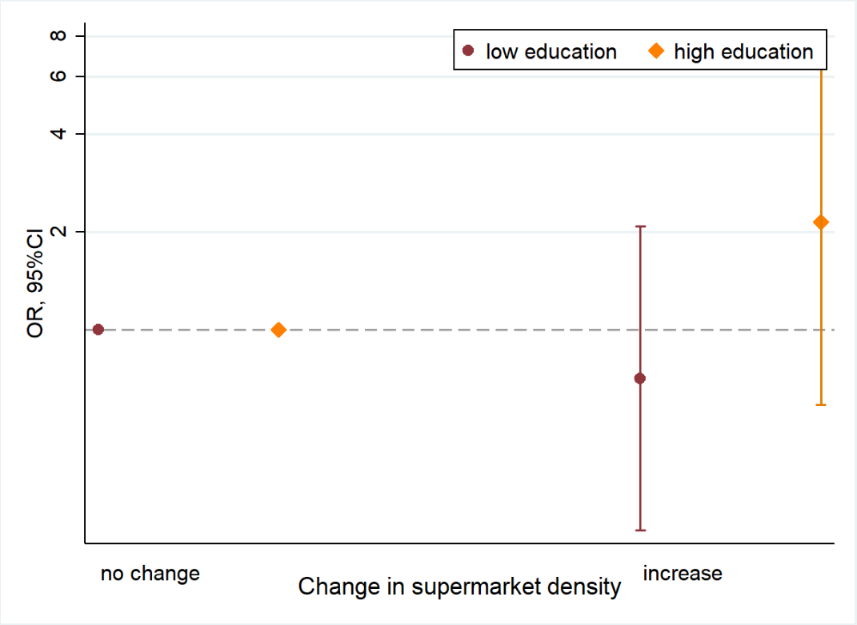


Interaction p=0.3610

Figure S5. Association between change in fruit and vegetable store density and odds of diabetes by change in supermarket density.


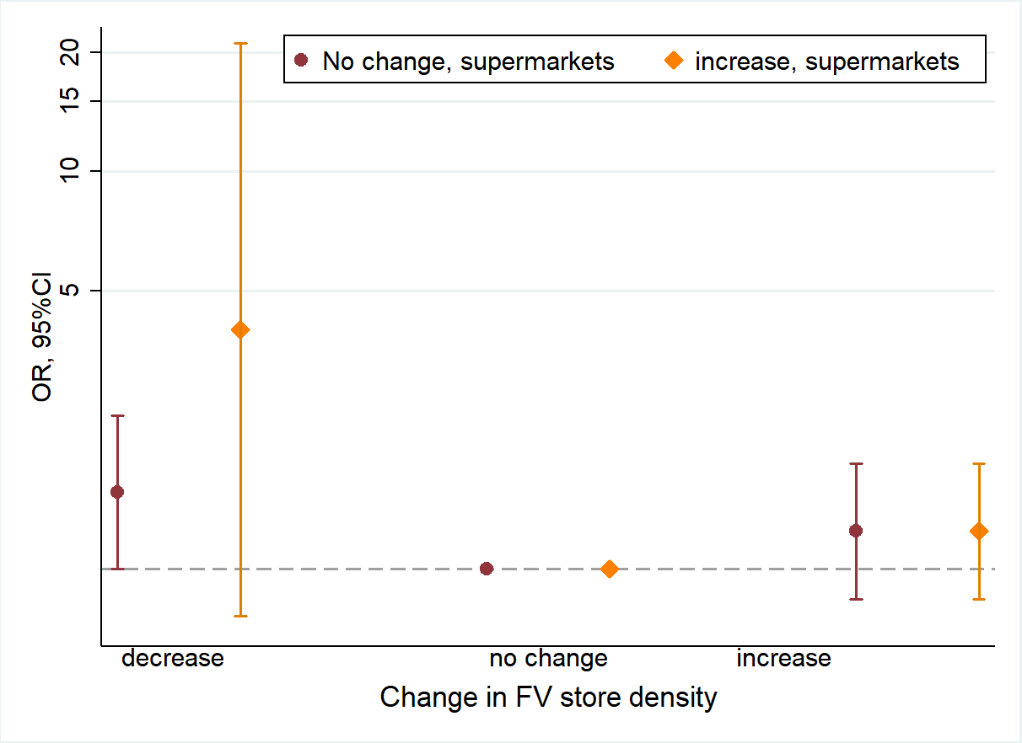


Interaction p=0.1681

References for suplemental material

1. Quistberg DA, Diez Roux AV, Bilal U *et al.* (2018) Building a Data Platform for Cross-Country Urban Health Studies: the SALURBAL Study. *Journal of urban health : bulletin of the New York Academy of Medicine*.
